# Pain response and symptoms in photorefractive keratectomy: mechanical de-epithelization compared with transepithelial ablation

**DOI:** 10.5935/0004-2749.20220026

**Published:** 2025-08-21

**Authors:** Priscila Rymer, Bernardo Kaplan Moscovici, Rachel Gomes, Braulio Couto, Paulo Schor, Mauro Campos

**Affiliations:** 1 Departamento de Oftalmologia, Escola Paulista e Medicina, Universidade Federal de São Paulo, São Paulo, SP, Brazil; 2 Universidade de Belo Horizonte, Belo Horizonte, MG, Brazil

**Keywords:** Pain, postoperative, Astigmatism, Myopia, Hyperopia, Photorefractive keratectomy, Laser, excimer/therapeutic use, Dor pós-operatoria, Astigmatismo, Miopia, Hiperopia, Ceratectomia fotorrefrativa, Laser de excimer/uso terapêutico

## Abstract

**Purpose:**

To compare postoperative pain and discomfort between mechanical and transepithelial photorefractive keratectomies.

**Methods:**

This prospective comparative study included 190 eyes of 95 patients with hyperopia (up to +4.00 D), astigmatism (up to -5.00 D), and myopia (up to -8.00 D) who underwent mechanical photorefractive keratectomy in one eye and transepithelial photorefractive keratectomy in the contralateral eye using Wavelight Allegretto EX500 excimer laser. The patients were unaware of the side treated with each technique. The interval between operations in the same patient was 15-30 days. Both eyes had similar refraction before surgery, with a maximum of 15-mm difference in ablation. Postoperative questionnaires were administered on days 1 and 7 to assess the patients’ level of discomfort (0=no discomfort to 5=extreme discomfort) with the following symptoms: pain, burning sensation, itchiness, tearing, photophobia, eye redness, foreign body sensation, and eyelid swelling. Patients were also asked about which method they preferred.

**Results:**

The sample consisted of 61 women (64.21%) and 34 men (35.79%). The mean (SD) patient age was 31.66 (6.69) years (range, 22-54 years). On postoperative day 1, the patients reported significantly less discomfort in terms of pain (1.9 ± 1.74 vs 2.5 ± 1.83; p=0.017), burning sensation (1.8 ± 1.56 vs 2.5 ± 1.68; p=0.004), tearing (2.3 ± 1.71 vs 3.1 ± 1.69; p=0.001), and foreign body sensation (1.9 ± 1.77 vs 2.5 ± 1.86; p=0.024) in the eye that received mechanical photorefractive keratectomy than in the eye that received transepithelial photorefractive keratectomy. No significant differences were found between the mechanical and transepithelial photorefractive keratectomies on postoperative day 7. Fifty-nine patients (62.10%) preferred mechanical photorefractive keratectomy, while 32 (33.68%) preferred transepithelial photorefractive keratectomy. Four patients (4.22%) expressed no preference.

**Conclusions:**

Our results showed that pain scores were significantly lower in the mechanical photorefractive keratectomy-treated eyes than in the transepithelial photorefractive keratectomy-treated eyes on postoperative day 1, which may have provided greater patient comfort after surgery and led patients to prefer the mechanical photorefractive keratectomy technique.

## Abstract (pt)

**Objetivo:**

Comparar a dor e o desconforto da ceratectomia fotorefrativa mecânica vs transepitelial.

**Métodos:**

Este estudo comparativo prospectivo incluiu 190 olhos de 95 pacientes com hipermetropia, astigmatismo e miopia que foram submetidos a ceratectomia fotorefrativa mecânica em um olho e ceratectomia fotorefrativa transepithelial no olho contralateral usando o laser excimer wavelight Allegretto EX500.O intervalo entre as operações no mesmo paciente foi de 15 a 30 dias. Ambos os olhos tiveram a refração similar antes da cirurgia, com uma diferença máxima de 15-mm na ablação. Os questionários pós-operatórios foram aplicados nos dias 1 e 7 para avaliar o nível de desconforto dos pacientes (0= sem desconforto para 5= desconforto extremo) com os seguintes sintomas: dor, sensação de queimação, coceira, lacrimejamento, fotofobia, vermelhidão dos olhos, sensação corpo estranho e inchaço das pálpebras. Os pacientes também foram questionados sobre qual método eles preferiram.

**Resultados:**

A amostra foi composta por 61 (64,21%) mulheres e 34 (35,79%) Homens. A idade média (SD) do paciente era 31.66 (6.69) anos. No primeiro dia pós-operatório, os pacientes relataram menos desconforto no olho que recebeu ceratectomia fotorefrativa mecânica do que ceratectomia fotorefrativa transepithelial (1,9 ± 1,74 vs 2,5 ± 1,83; *p*=0,017), sensação de queimação (1,8 ± 1,56 vs 2,5 ± 1,68; *p*=0,004), lacrimejamento (2,3 ± 1,71 vs 3,1 ± 1,69; *p*=0,001), e sensação corpo estranho (1,9 ± 1,77 vs 2,5 ± 1,86; *p*=0,024). Não foram encontradas diferenças significativas entre ceratectomia fotorefrativa mecânica e ceratectomia fotorefrativa transepithelial no dia 7 pós-operatório. Cinquenta e nove pacientes (62,10%) preferiram ceratectomia fotorefrativa mecânica, enquanto 32 (33,68%) preferiram ceratectomia fotorefrativa transepithelial. Quatro pacientes (4,22%) não expressaram nenhuma preferência.

**Conclusões:**

Nossos resultados mostraram que os escores de dor foram significativamente menores nos olhos tratados pelo ceratectomia fotorefrativa mecânica do que nos olhos tratados com ceratectomia fotorefrativa transepithelial no primeiro dia pós-operatório, o que pode ter proporcionado maior conforto do paciente após a cirurgia e os levou a ter uma preferência pela técnica ceratectomia fotorefrativa mecânica.

## INTRODUCTION

Photorefractive keratectomy (PRK) is a technique widely used for the treatment of ametropia and consists of removing the epithelial layer of the cornea, followed by ablation of the underlying stroma with excimer laser^(1)^. Nevertheless, it is associated with slow visual recovery and significant postoperative pain^(1)^.

Epithelial removal and stromal ablation promote corneal nerve injury, with consequent release of cytokines, growth factors, and metalloproteases that play a critical role in corneal wound healing. Therefore, pain is more severe on the first postoperative days owing to exposure of the corneal nerves and the release of the cytokines and other factors after surgery^(1-6)^. Other common symptoms include tearing, eye redness, foreign body sensation, burning sensation, itchiness, photophobia, and eyelid swelling^(1-6)^.

The epithelial layer can be removed using a spatula, 5%-25% diluted alcohol for 10-20 seconds, or excimer laser (transepithelial). The advantages of transepithelial PRK (tPRK) include faster and easier de-epithelization, which leads to less stromal dehydration and faster epithelial recovery^(2)^. It uses an epithelial thickness profile similar to that used in phototherapeutic keratectomy (PTK) to remove the epithelium, on the basis of the average epithelial thickness of a population of normal eyes (53 mm centrally and 58 mm at the 6-mm periphery) as measured with different methods such as optical coherence tomography and confocal microscopy^(7-13)^. All the methods of corneal epithelial cell removal are effective, but only a few studies have compared postoperative symptoms and patient satisfaction between the techniques^(14,15)^.

The present study aimed to compare postoperative pain and discomfort between the mechanical PRK (mPRK) and tPRK techniques.

## METHODS

This prospective comparative study was conducted in the Department of Ophthalmology of the Federal University of Sao Paulo, Brazil, and included 190 eyes of 95 patients with hyperopia, astigmatism, and myopia who underwent mPRK in one eye and tPRK in the contralateral eye. Written informed consent was obtained from all the patients prior to their inclusion in the study. The study was approved by our institutional research ethics committee (approval No. 556234).

Eligible participants were all patients aged 20 to 60 years with a minimum corneal pachymetric value of 450 mm, myopia of up to -8.00 D, astigmatism of up to -5.00 D, and hyperopia of up to +4.00 D. The patients were excluded if they had autoimmune diseases, other eye diseases, diabetes, previous ocular surgery, or history of corneal infection; if they used oral medications that cause dry eye (isotretinoin, steroids, and antidepressants); and if they had clinical or topographic evidence of keratoconus. One eye in each patient was randomly chosen to undergo mPRK; and the other eye, to undergo tPRK. There was no preference as to which eye was operated first; this choice was random, so half of the first operated eyes underwent mPRK, and the other half underwent tPRK. The patients were unaware of which side was treated with each technique. They were instructed to discontinue wearing contact lenses 3 weeks before surgery.

All surgical procedures were performed by the same surgeon (B.K.M.) using the Wavelight Allegretto EX500 excimer laser system (Alcon Laboratories, Fort Worth, TX, USA). The interval between the first and second surgeries on the same patient was 15 days. Both eyes had similar refractions before surgery, with a maximum of 15-mm difference in ablation. Treatment was performed according to the patient’s refraction and laser nomogram. The epithelial debridement zone was 9 mm. Mechanical epithelial removal was performed with a blunt spatula, while transepithelial removal was performed using the PTK mode of the excimer laser system. All the procedures were performed with a 6.5-mm stromal ablation zone for all the patients. Immediately after ablation, mitomycin C 0.02% was applied to the ablated surface for 30 seconds, followed by irrigation with 20 mL of balanced salt solution. At the end of each procedure, a drop of moxifloxacin 0.5% + dexamethasone 0.1% (Vigadexa; Alcon Laboratories, Fort Worth, TX, USA) was administered to the treated eye, and a bandage contact lens was placed^(16,17)^. Bandage contact lens of the same model and brand were used in all the cases (Biomedics 55; CooperVision, Lake Forest, California, USA).

After operation, all the patients were instructed to apply a drop of moxifloxacin 0.5% + dexamethasone 0.1% (Vigadexa) 4 times a day for 7 days, ketorolac tromethamine 0.4% (Acular LS; Alcon Laboratories, Fort Worth, TX, USA) 3 times a day for 3 days, and ocular lubricant (Hyabak; Thea, Clermont-Ferrand, France) 4 times a day for 30 days. After the initial 7 postoperative days, prednisolone acetate 0.12% (Pred Mild; Allergan Inc., Irvine, CA, USA) eye drops were administered 4 times a day for the next 3 postoperative weeks. Oral pain medication was also prescribed to be taken as needed in the first postoperative week (dipyrone, 500 mg 4 times a day). Patients who misused the postoperative medication would be excluded from the study, but none had to be excluded.

Postoperative questionnaires were administered on days 1 and 7 to assess patients’ symptoms according to the routine of our institution. The patients were asked to rate on a scale from 0 (no discomfort) to 5 (extreme discomfort) their level of discomfort with the following symptoms: pain, burning sensation, itchiness, tearing, photophobia, eye redness, foreign body sensation, and eyelid swelling. They were also asked about which method they preferred. The study evaluated the symptoms on the first day to assess them when they are most severe and on seventh day to assess their recovery and improvement.

The pain response and other symptoms in the PRK group were measured using a self-administered questionnaire, with possible scores ranging from 0 (minimum value) to 5 (maximum symptom). The patients self-rated their pain response and other symptoms on the 5-point scale on the first and seventh days after surgery. We used non-parametric statistical tests, as we tested the normality of the quantitative variables of the main outcome using the Kolmogorov-Smirnov test and concluded that a normality distribution could not be assured. We used the Mann-Whitney test, considering a level of significance of 5%. We used the SPSS version 20 software for all statistical analyses, including graphs.

## RESULTS

The sample consisted of 61 women (64.21%) and 34 men (35.79%). The mean (SD) patient age was 31.66 (6.69) years (range, 22-54 years). All 95 patients (190 eyes) completed the questionnaires on postoperative days 1 and 7.

**Figure 1:**
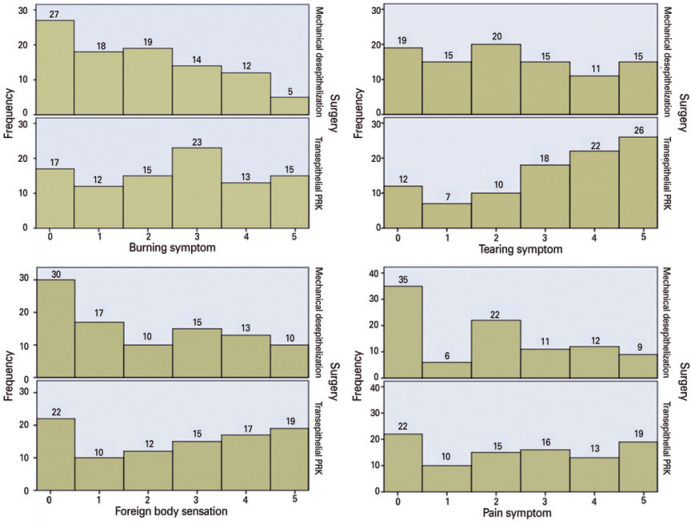
Histograms of the symptom scores on postoperative day 1 (0= no discomfort to 5= extreme discomfort).

Table 1 shows the mean discomfort scores reported by the patients on day 1. The patients reported significantly less pain in the eye that received mPRK than in the eye treated with tPRK (1.9 ± 1.74 vs 2.5 ± 1.83; p=0.017). While half of the eyes that received tPRK had pain scores of 3, 4, or 5 (50.5%), only one-third of the eyes treated with mPRK had scores ≥3 (33.7%; Figure 1). The patients also reported significantly less discomfort in terms of burning sensation (1.8 ± 1.56 vs 2.5 ± 1.68; p=0.004), tearing (2.3 ± 1.71 vs 3.1 ± 1.69; p=0.001), and foreign body sensation (1.9 ± 1.77 vs 2.5 ± 1.86; p=0.024) with mPRK than with tPRK. Likewise, the tPRK-treated eyes were more frequently rated with scores of 3, 4, and 5 for burning sensation (53.7% vs 32.6%; Figure 1), tearing (69.5% vs 43.1%; Figure 1), and foreign body sensation (53.7% vs 40.0%; Figure 1), respectively. For the first eye, no significant difference was found between the patients who received mPRK and those who received tPRK in relation to the pain score (1.7 ± 1.54 vs 2.0 ± 1.39; *t*-test p=0.281). However, for the second eye the results suggest that the patients who received mPRK had less pain discomfort than those who received tPRK (2.0 ± 1.36 vs 2.6 ± 1.68; *t*-test p=0.086).

**Table 1:** Mean discomfort scores on postoperative day 1

Symptom	Mechanical PRK mean (SD)	Transepithelial PRK mean (SD)	p^*^
Burning sensation	1.8 (1.56)	2.5 (1.68)	0.004
Itchiness	0.8 (1.16)	1.1 (1.38)	0.313
Tearing	2.3 (1.71)	3.1 (1.69)	0.001
Pain	1.9 (1.74)	2.5 (1.83)	0.017
Photophobia	3.0 (1.49)	3.3 (1.52)	0.122
Eye redness	1.5 (1.54)	1.8 (1.61)	0.253
Foreign body sensation	1.9 (1.77)	2.5 (1.86)	0.024
Eyelid swelling	1.5 (1.53)	1.8 (1.81)	0.297

PRK= photorefractive keratectomy; SD= standard deviation.

* Mann-Whitney test.

PRK= photorefractive keratectomy.

On postoperative day 7, we found no statistically significant differences in any symptoms between the mPRK and tPRK techniques (Table 2). Regarding the preferred technique, 59 patients (62.10%) preferred mPRK, while 32 (33.68%) preferred tPRK. Four patients (4.22%) expressed no preference.

**Table 2:** Mean discomfort scores on postoperative day 7

Symptom	Mechanical PRK mean (SD)	Transepithelial PRK mean (SD)	P^*^
Burning sensation	0.4 (0.85)	0.7(1.25)	0.293
Itchiness	0.4 (0.82)	0.5 (0.86)	0.433
Tearing	0.6 (1.15)	0.9(1.35)	0.195
Pain	0.5 (1.06)	0.6 (1.23)	0.710
Photophobia	1.4(1.42)	1.8 (1.50)	0.082
Eye redness	0.4 (0.85)	0.6 (1.14)	0.156
Foreign body sensation	1.0(1.39)	1.0(1.40)	0.679
Eyelid swelling	0.3 (0.76)	0.4(1.07)	0.852

PRK= photorefractive keratectomy; SD= standard deviation.

* Mann-Whitney test.

## DISCUSSION

Pain and discomfort in the immediate postoperative period are a challenging but common problem in PRK. In the present study, the comparisons revealed that mPRK was superior to tPRK in terms of the patients’ pain experience and level of discomfort with tearing, foreign body sensation, and burning sensation on postoperative day 1, with no significant differences between the two techniques on postoperative day 7. The patients also expressed a preference for the mPRK technique. The interval between surgeries was 15 days, as it is the standard procedure in our service.

Lee et al.(2) were the first to evaluate epithelial healing and postoperative pain after PRK using three epithelial removal techniques (mechanical, 20% diluted alcohol, and excimer laser-assisted), but no significant differences were found between the groups after 6 months. Buzzonetti et al.^(18)^ performed tPRK using the Nidek CXIII excimer laser and reported a mean (SD) pain score of 3.0 (1.2) (range, 1-6) after 3 months. Mohebbi et al.^(4)^, using the Technolas 217P excimer laser, evaluated the potential role of sub-basal nerve plexus density in the occurrence of pain after PRK, but no relationship could be established.

Previous studies of PRK postoperative symptoms, for the most part, reported less pain and better healing with the tPRK technique^(19-28)^. These studies, however, used laser systems different from that used in the present study. The authors are unaware of a previous study that compared postoperative pain and other symptoms between mPRK and tPRK performed with the Wavelight Allegretto EX500 excimer laser system.

Wang et al.^(3)^ compared laser-assisted subepithelial keratectomy (LASEK) and tPRK using the Schwind ESIRIS excimer laser and reported significantly less pain in the LASEK group on postoperative day 1 (3.2 ± 1.88 vs 4.43 ± 1.61, p=0.008), which increased slightly on postoperative days 2 and 3 but did not differ from that in the tPRK group. Korkmaz et al.^(20)^ also compared LASEK and tPRK using the Schwind ESIRIS excimer laser and reported that the mean time to epithelial healing was significantly longer after LASEK (4.00 ± 0.43 vs 3.17 ± 0.6 days, p<0.05), but the mean subjective pain score on day 1 was significantly higher after tPRK (3.75 ± 0.87 vs 1.92 ± 1.83, p<0.05). After day 1, the mean pain scores were similar in the two groups^(20)^. Kaluzny et al.^(21)^ compared alcohol-assisted PRK and tPRK performed with the Schwind AMARIS excimer laser and reported similar mean pain scores immediately after surgery in the two groups (4.78 ± 2.65 and 4.59 ± 2.85, respectively; p=0.85). In addition, no significant differences were observed in pain intensity during the first postoperative days (4.46 ± 2.54 vs 4.51 ± 2.36, respectively; p=0.86). When the patients were asked about their overall satisfaction with the procedure, 86.25% were highly satisfied after tPRK as compared with the 88.24% who were highly satisfied after alcohol-assisted PRK (p=0.46)^(21)^.

However, the different results from our present findings were obtained in studies that compared mPRK and tPRK performed with the Schwind AMARIS excimer laser system. Celik et al.^(5)^ reported that the mean time to complete epithelial healing was significantly longer after mPRK than after tPRK (3.76 ± 0.43 vs 2.19 ± 0.39 days, p<0.001) and that pain scores were higher in mPRK-treated eyes than in tPRK-treated eyes on postoperative day 1 (5.59 ± 0.54 vs 3.95 ± 0.58, p<0.001) and day 3 (3.38 ± 0.73 vs 2.80 ± 0.45, p<0.001). Naderi et al.^(22)^ also found significantly higher pain scores at 24 hours after mPRK (3.3 ± 0.71) than after tPRK (2.30 ± 0.56; p=0.04). Fadlallah et al.^(23)^ reported faster epithelial healing after tPRK than after mPRK (2.5 ± 0.6 vs 3.7 ± 0.8 days, p=0.01) and less pain at 48 hours (2.00 ± 1.39 vs 4.12 ± 1.40, p=0.02).

The literature shows that tPRK causes fewer symptoms than mPRK in the studies that used the Schwind platform. However, the studies did not report whether the de-epithelialized area was the same in both techniques. In the present study, both techniques had the same de-epithelialized area using the Wavelight Allegretto EX500 platform. This allowed us to hypothesize that the tPRK group would have more symptoms than the mPRK group owing to the energy used for this process.

El Rami et al. studied pain in sequential LASIK surgeries and found greater pain in the second surgery, without defining any specific cause other than psychological or unknown factors. However, because our study had an interval of 15 days between surgeries and was randomized, we think this could have little effect on our results^(29)^.

Among the first eyes treated, those treated with tPRK had worse pain, but the difference was not statistically significant. The eyes treated next had more pain than those that were treated first, similarly in the report of El Rami et al.^(29)^. The difference between tPRK and mPRK was statistically significant (more pain in tPRK). We wonder whether this was influenced by any psychological factor or a residual inflammatory response from the first surgery. Our study has few limitations. First, we only evaluated pain on the first and seventh day after operation and not daily; however, we asked the patients which technique they preferred in relation to pain and symptoms, so we covered the symptoms in the whole initial postoperative period. Other limitations were that we did not use the visual analog scale to assess pain and a validated questionnaire, but because our results were significant, we wonder if this would make a difference in the final results. Finally, we used some studies with alcohol-assisted PRK in the discussion because only few studies compared mPRK with tPRK.

In conclusion, we found that on postoperative day 1, pain and discomfort with tearing, foreign body sensation, and burning sensation showed significant differences between mPRK and tPRK performed with the Wavelight Allegretto EX500 excimer laser. In the mPRK-treated eyes, the pain scores were lower, which may have provided greater patient comfort after surgery and led them to prefer the mPRK technique. Although both mPRK and tPRK are effective techniques, further studies with similar de-epithelialized areas in both techniques are needed to evaluate post-PRK symptoms and discomfort. Future studies should also assess the new Wavelight Allegretto Streamlight tPRK laser technology platform.
